# Construction and evaluation of a bioluminescent *Pseudomonas aeruginosa* reporter for use in preservative efficacy testing

**DOI:** 10.1099/mic.0.001072

**Published:** 2021-08-12

**Authors:** Laura Rushton, Denise Donoghue, Matthew Bull, Peter Jay, Eshwar Mahenthiralingam

**Affiliations:** ^1^​ Cardiff School of Biosciences, Cardiff University, Cardiff, UK; ^2^​ Unilever R&D Port Sunlight, Wirral, Merseyside, UK; ^†^​ Present address: Public Health Wales Microbiology, University Hospital Wales, Cardiff, Wales, UK

**Keywords:** bioluminescence, *Luxreporter*, preservative efficacy testing, *Pseudomonas aeruginosa*, rapid microbiology testing

## Abstract

Preservative efficacy testing (PET) is a fundamental practice in industrial microbiology used to ensure product shelf-life and quality. To improve on current growth-based PET, bioluminescence was evaluated as a real-time bacterial viability indicator using *Pseudomonas aeruginosa *. Random mutagenesis of an industrial *P. aeruginosa* strain with a promoter-less *luxCDABE* mini-Tn5 was used to select a stable reporter (LUX12H5) with an un-altered growth and preservative susceptibility phenotype. Bioluminescence and viability were measured with and without preservatives (isothiazolinones, phenoxyethanol, and dimethyl dimethylol hydantoin) and an antibiotic comparator (ciprofloxacin). In the absence of antimicrobials, a good correlation between bioluminescence and viability (r^2^=0.92) was established. However, metabolic inhibition by isothiazolinone preservatives caused a rapid decline in light output that did not correlate to a reduced viability. Conversely, after ciprofloxacin exposure, the decline in viability was greater than that of bioluminescence. A positive attribute of the bioluminescence was the early detection of metabolic recovery and re-growth of preservative injured bacteria. Overall, while initial bioluminescence read-outs were less suited to current PET requirements, it shows promise as an early, direct indicator of bacterial regrowth in the context of long-term evaluation of preservative efficacy.

## Introduction

Antimicrobial preservatives are often incorporated into raw materials and finished industrial products to inhibit proliferation of spoilage or pathogenic microorganisms, which could be introduced during manufacturing or through consumer use. Several factors may influence the activity of the preservative system; consequently, it is difficult to predict its effectiveness in complex formulations and assurance is required by means of a preservative efficacy test (PET) [1]. Standardised methods of PET are published in the European Pharmacopoeia, ISO global standards (11930) and by trade associations such as the Personal Care Products Council. Fundamentally, PET protocols require test samples to be inoculated with microorganisms representative of spoilage or pathogenic contaminants. The inoculated product is then sampled at defined time intervals and microbial survival monitored by re-growth on agar. Preservative systems must meet specified reductions in the viable counts for a range of microorganism over a period of 28 days [2] to comply with PET standards. Current culture-based PET protocols are labour intensive and may lack sensitivity since they require the use of neutralisers to quench preservatives and dilution steps, both of which may diminish microbial numbers and reduce accuracy. Rapid and direct methods of determining microbial viability such as impedance or adenosine triphosphate (ATP) bioluminescence can be used as alternatives to colony counting [1, 3, 4] but have inherent limitations in relation to PET. ATP-bioluminescence for example requires microbial capture and cell lysis to release ATP, with measurement of the bioluminescence generated using luciferin-luciferase reagents [1]. Preservatives and product ingredients may impact greatly on the exogenous bioluminescence reaction and accuracy is also dependent on efficient ATP extraction [5].

*In vivo* or whole-cell bioluminescence offers an alternative rapid approach to monitoring the viability of target microorganisms in PET. A well characterised and frequently used bacterial bioluminescent system comes from the terrestrial Gram-negative bacterium *Photorhabdus luminescens* [6]. In the *P. luminescens* luciferase (*lux*) system, all of the genes required for bacterial bioluminescence are organised in a single operon *luxCDABE* [7]; therefore, the addition of an exogenous substrate is not necessary. Bacterial luciferases catalyse the oxidation of reduced flavin mononucleotide (FMNH_2_) and a long-chain aldehyde by molecular oxygen, resulting in the emission of light at 490 nm [8]. As the reaction is dependent on the reducing power of FMNH_2_ only metabolically active cells can emit light. These systems have been developed into a wide and versatile range of tools for molecular microbiology [6, 9]. In application, recombinant bacteria engineered to express *lux* genes are proven to be sensitive reporters of the real-time effect of antibiotics [10, 11] and biocides [12] on bacterial growth, and are sensitive indicators of cellular viability in experimental systems [9]. Encouragingly, a recent study by Shah and Naseby [13] reported quantification of *Pseudomonas aeruginosa* by whole-cell bioluminescence was to be at least equivalent if not better than traditional plate counting and the ATP determination methods.

The main objective of this study was to explore the applicability of whole-cell bioluminescence for PET in the personal care industry. *P. aeruginosa* was selected as a suitable test species as it is an objectionable, frequently encountered Gram-negative bacterial industrial contaminant [14–16] that is recommended for inclusion by standardised PET protocols [17]. In this study, a stable bioluminescent reporter *P. aeruginosa* strain was created by random transposon mutagenesis using mini-Tn5 containing a promoter-less *luxCDABE* operon [18], a system successfully used previously as a reporter of gene expression in the species [19]. The bioluminescence reporter was also constructed within an industry relevant, genome-sequenced *P. aeruginosa* strain, RW110, originally isolated from a contaminated home cleaning product [20]. This approach provided multiple advantages: chromosomal integration of the *lux* genes generated a single-copy reporter construct with greater stability than plasmid-based systems, fitting a fundamental requirement that PET is conducted over a period of weeks; screening a mutant library identified derivatives with stable and high bioluminescence, suitable as *lux* reporters in sensitive assays where low viable cell numbers are detected; and a bespoke reporter could be constructed in a valid industrial *P. aeruginosa* strain which had been used historically in PET. We successfully implemented this novel *P. aeruginosa lux* biosensor selection process, and used the resultant reporter strains to evaluate the relationship between bioluminescence and viability as a rapid and direct method of PET.

## Methods

### Chemicals and growth media

Tryptone soya medium (agar, TSA; broth, TSB) and Lysogeny Broth (LB) medium were obtained from Oxoid Ltd. Tetracycline (Tc) and ciprofloxacin (CIP) antibiotics were supplied by Sigma-Aldrich; dimethyl sulfoxide (DMSO) and other analytical grade chemicals were supplied by Fisher Scientific. A minimal basal salts growth medium (BSM) was prepared as described by Rushton *et al*. [21] without casamino acids. A 3 : 1 cosmetics grade blend of chloromethylisothiazolinone and methylisothiazolinone (CMIT/MIT), and methylisothiazolinone (MIT) were obtained from Dow Chemical Company, USA; dimethyl dimethylol hydantoin (DMDMH) from Lonza, UK; benzisothiazolinone (BIT) and phenoxyethanol (PH) from Clariant International Ltd, Switzerland. Preservative stock solutions were prepared (in sterile distilled water or DMSO for PH) on the day of use and diluted as required.

### Bacterial strains and construction of bioluminescent *P. aeruginosa*

*P. aeruginosa* RW110 an industrial isolate used routinely in PET [20] was utilised in this study. Revival of the frozen isolate was performed on TSA plates at 30 °C. *Escherichia coli* S17-1 λpir donor strain carrying pUTmini-Tn5-*luxCDABE*-Tc was grown on LB containing 10 µg ml^−1^ Tc at 37 °C. Overnight liquid cultures (3 ml) were inoculated and incubated for 18 h with aeration at an appropriate temperature. Liquid cultures were standardised to an optical density (OD_630_) of one, which equated to approximately 10^8^ c.f.u. ml^−1^, and were diluted as required. Bacterial stocks were stored at −80 °C in TSB or LB containing 8% v/v DMSO. All experiments were performed as two biological replicates with a range of technical replicates (two to six). The mini-Tn5-*luxCDABE* transposon was delivered into the recipient *P. aeruginosa* RW110 by conjugal mating with the *E. coli* donor as described by Lewenza *et al.* [19] with the following modifications. Recipient cells were grown at 30 °C and heat shocked for 2 min at 42 °C prior to biparental mating; donor and recipient were mixed at a ratio of 1 : 1 on a nitrocellulose filter and incubated on LB agar containing 10 mM MgSO_4_ for 24 h at 37 °C. To select *P. aeruginosa* transconjugants, the mixture was re-suspended in TSB and plated onto TSA plates containing 200 µg ml^−1^ Tc. Transconjugants were picked into white 96-well plates containing 200 µl TSB and 50 µg ml^−1^ Tc, cultured for 24 h on an orbital shaker at 30 °C. Bioluminescence was measured before the addition of DMSO (final concentration 8 % v/v) and plates were frozen at −80 °C for storage.

### Bioluminescent measurements

Bioluminescent measurements were performed in flat-bottom white 96-well microtitre plates (Greiner, BioOne) using a Tecan Infinite M200 PRO luminometer (Labtech International, UK), with a 1 s integration time. Microtitre plates were dark treated prior to loading and measurements were delayed for 15 min to allow photons trapped in the plastic to dissipate. The instrument level of background ranged from 0 to 20 relative light units (RLU); therefore, a threshold for a bioluminescent light measurement was set as ≥20 RLU. For test measurements, the 200 µl samples were staggered (minimum of one well separation) across the microtitre plate to reduce crosstalk between wells; non-test or control wells contained 200 µl TSB. The mean RLU of the sample blank was subtracted from each test well and the mean RLU of biological replicates, each with duplicate technical replicates, was then determined for each sample. Observations were first made 1 h after sample inoculation as the instrument was not fitted with an injector system.

### Screening bioluminescent *P. aeruginosa* for suitability in PET

*P. aeruginosa* RW110 transposon mutants suitable for application in PET were identified by screening with the following criteria: a high level of bioluminescence (≥5 Log_10_ RLU); a stable transcriptional gene fusion maintained in the absence of tetracycline; unaltered growth characteristics; prototrophy; and unchanged preservative susceptibility. The 94 highest light emitters were selected from the initial bioluminescent measurement of the transconjugants and sub-cultured in a white 96-well microtitre plate with an optically clear bottom (Greiner). The wild-type isolate and a sample blank were included on the plate. To determine the stability of transcriptional gene fusion, the sub-group of mutants were replica plated and sub-cultured three times (24 h cultures) in TSB without tetracycline. Measurements of bioluminescence were normalised to the OD_630_ of each well (i.e. RLU/ OD_630_) and mutants were ranked in order of light levels. Growth characteristics of the sub-group were determined by turbidometric analysis by transferring 5 µl of an overnight starter culture into a 96-well microtitre plate containing 195 µl of TSB (in triplicate). Growth at 30 °C was monitored over 48 h using a Bioscreen MBR (Labsystems, Finland) as described [21]. The length of lag-phase (hours), growth rate represented by the maximum slope (*µ*), and maximal growth (OD_420-580_) of each mutant were determined using R statistical software [22] and the Grofit package [23] and compared to the wild-type. To confirm prototrophy, an 18 h culture of the mutants were replica plated onto minimal media (BSM) with 0.4 % (w/v) glucose as the sole carbon source. The susceptibility of the wild-type and mutants to five preservatives frequently used in industry was determined by agar dilution. Then 18 h cultures were replica plated onto TSB (with 3 % w/v purified agar (Oxoid), to reduce the spread of colonies) containing CMIT/MIT at 0.000313–0.005 %, DMDMH at 0.016–0.25 %, MIT at 0.0012–0.019 %, BIT at 0.002–0.038 % and PH at 0.125–0.75 % active. The minimum inhibitory concentration (MIC) was then read after 24 h incubation at 30 °C. The bioluminescence of the top five mutants fulfilling all the selection criteria above, were monitored during culture in TSB over 48 h and normalised to viable counts (c.f.u. ml^−1^) that were determined in parallel (i.e. RLU/ c.f.u. ml^−1^).

### Mapping the genomic location of the Tn5 insertion

The insertion sites of the bioluminescent transposon mutants were mapped by genome sequence analysis as follows. Genomic DNA was extracted from pure overnight cultures of the wild-type and the five highest performing mutants, (Table 1) using the Maxwell16 instrument (Promega) and a Maxwell 16 Tissue DNA purification kit, according to the manufacturer’s instructions. After RNase A treatment, the DNA was quantified using a Qubit 3.0 fluorometer and eluted to a normalised concentration of 50 ng µl^−1^ in 10 mM Tris-HCl. DNA was sent for library preparation and sequencing on an Illumina MiSeq at the Earlham Institute (UK). The resulting reads were trimmed with trim-galore v 0.3.5 [24], overlapping reads were extended using FlaSh v1.2.2 [25] and assembled using SPAdes v3.0.0 [26]. Annotation of the assembly and prediction of gene function was carried out using Prokka v1.09 [27]. To identify the location of the min-Tn5 insertion, a local blast database of the genome assemblies was created and compared to the reference genomes *P. aeruginosa* PA01 (GCF_000006765.1) and *P. aeruginosa* PA14 (GCF_000404265.1) using a command line nucleotide (Blastn) search (blast+v2.2.26). Comparison crunch files were generated and Easyfig (v2.1.0) [28] utilised to generate comparative synteny line plots between the genome sequences of *P. aeruginosa* PA01 and the *P. aeruginosa* RW110 LUX12H5 mutant. To confirm each mini-Tn5 was identical in sequence, a local blast database of the mini-Tn5-*luxCDABE* sequences from the mutants (Table 2) was also created and compared by Blastn to the mini-Tn5-*luxCDABE* sequence of mutant LUX12H5 as a reference.

**Table 1.** The features of the five highest performing *Pseudomonas aeruginosa luxCDABE* mutants

Mutant ID	Bioluminescence/ OD		Growth characteristics						Preservative MIC (% active)				
	Revival	Sub-culture	Growth rate µ (±STDEV)		Lag phase hours (±STDEV)		Maximum growth OD (±STDEV)		MIT	CMIT/MIT	BIT	PH	DMDMH
Wild-type	0	0	0.259	(±0.008)	1.106	(±0.104)	1.525	(±0.004)	0.00468	0.0025	0.01875	0.5	0.0625
1 C5	534 114	508 318	0.265	(±0.008)	1.123	(±0.104)	1.525	(±0.004)	0.00468	0.0025	0.01875	0.5	0.0625
12 H8	308 587	496 781	0.242	(±0.006)	0.839	(±0.084)	1.111	(±0.003)	0.00468	0.0025	0.009375	0.5	0.0625
1 G4	522 108	442 315	0.23	(±0.004)	0.913	(±0.059)	1.615	(±0.003)	0.00468	0.0025	0.01875	0.5	0.0625
12 H5	313 099	397 337	0.231	(±0.005)	0.822	(±0.089)	1.616	(±0.003)	0.00468	0.0025	0.009375	0.5	0.0625
1D2 *	395 773	392 670	0.168	(±0.003)	4.304	(±0.101)	1.547	(±0.003)	0.00468	0.0025	0.009375	0.5	<0.015625

∗Mutant excluded as it failed to meet the selection criteria.

OD, optical density at 630 nm; MIC, minimum inhibitory concentration; MIT, methylisothiazolinone; CMIT/MIT, chloromethylisothiazolinone and methylisothiazolinone blend; BIT, benzisothiazolinone; PH, phenoxyethanol; DMDMH, dimethyl dimethylol hydantoin.

Mutants ranked in order of bioluminescence/optical density after three sub-cultures in the absence of tetracycline selection. Bioluminescence/optical density of a 24 h culture of the revived master plate shown. Bioluminescence measured as relative light units.

**Table 2.** Genomic location of the mini-Tn5-*luxCDABE* insertion within the five highest performing *P. aeruginosa* mutants

Mutant ID	Length (bp)	PA01 Locus tag	Predicted function	Accession no.	Identity (%) to PAO1 genome
LUX 12H5	359	PA2277	ArsR protein	AAG05665.1	96.94
LUX 12H8	359	PA2277	ArsR protein	AAG05665.1	96.94
LUX1C5	1289	PA0534	FAD-dependent oxidoreductase	AAG03923.1	98.68
LUX1G4	1289	PA0534	FAD-dependent oxidoreductase	AAG03923.1	98.68
LUX 1C2	378	PA4659	Probable transcriptional regulator	AAG08046.1	99.74

*Homolog in *P. aeruginosa PAO1*.

### Bioluminescence assay range and limits of detection

The relationship between light output and viability was firstly assessed in the absence of antimicrobial. Overnight cultures of the selected reporter mutant were pelleted via centrifugation (1400 ***g***, 10 min) and resuspended in half the volume of fresh medium. A dilution series of the concentrated suspension was prepared in TSB and bioluminescence measured as described above. Viable cells were enumerated by serial dilution and plate count.

### PET

The following preservatives, commonly used in industry, were evaluated at concentrations corresponding to the maximum EU regulated levels for use in rinse-off personal care products [29]. CMIT/MIT blend 6.25e-06 to 0.0015 % w/w active, DMDMH at 0.0015625 to 0.25 % w/w active (below the max regulated level of 0.6 % active), and PH at 0.125 to 1 % v/v active. The PET procedure was modified from standard Pharmacopoeia guidelines as follows. Overnight cultures of the bioluminescent reporter strain were prepared and diluted as described above. The test medium was diluted (1 : 1) with bacterial culture, to achieve the final preservative test concentration and a starting inoculum of approximately 10^6^ c.f.u. ml^−1^, as is standard for PET. Test samples (5 ml) were incubated statically at 28 °C for 14 days in the presence of CMIT/MIT, and 21 days in the presence of DMDMH, to align with PET timelines. Test cultures were sampled after 1, 6, 24, 48, 168, 336 and 504 h, to measure bioluminescence and microbial survival in parallel. For PH, test samples were incubated as previously described for 24 h and assessed at 0, 1 and 24 h. Bioluminescence was measured directly without preservative neutralisation as described above. The viability of neutralised samples was determined in parallel. Preservative activity was quenched prior to plating by a ten-fold dilution in a 0.1 % peptone, 2 % tween neutralising solution. The efficiency of neutralisation was determined prior to experimentation, as described by Lear *et al.* [30].

### Ciprofloxacin challenge

The MIC of ciprofloxacin was determined by agar dilution method [31]. The effects of concentrations ranging four times higher and lower than the MIC on light output and viability were then determined. The preparation, inoculation and measurements of the test samples mirrored that of the preservative challenge over 1 week. With the exception that samples were diluted in saline and plated on TSA plates containing 1 % magnesium chloride to neutralise the antibiotic effect and aid recovery of survivors.

### Statistical analysis

To determine the relationship between bioluminescence (RLU) and viable count (c.f.u. ml^−1^) readings data were logarithmically (Log_10_) transformed, plotted against each other and linear regression analysis of the mean of biological replicate experiments performed (Microsoft Excel, 2010). Growth curve analysis was conducted using R statistical software [22] and the Grofit package.

## Results

### Construction and selection of *P. aeruginosa* suitable for PET

In total, 1111 *luxCDABE* mutants were randomly screened and evaluated as reporters. Their bioluminescence after initial culture (with tetracycline) ranged from undetectable (*n*=4) to 6 Log_10_ RLU (*n*=20). Mutants with high levels of bioluminescence, at ≥5 Log_10_ RLU (*n*=367), were ranked in order of light emission, and the top 94 were further characterised to identify those suitable for application in PET. The bioluminescence, growth characteristics and preservative MIC of this 94 sub-group was measured; data for the top five *lux* reporters is shown in Table 1, with the remaining 89 provided in the supplementary material (Table S1, available in the online version of this article). After three sub-cultures in the absence of tetracycline, 39 % of the 94 sub-group were deemed unsuitable, due to inconsistent light emission (bioluminescence decreasing to less than 4 Log_10_ RLU for cultures with an OD_630_ >1). The majority of mutants reached a maximal growth (OD_630_ ≥1.1) and a growth rate (*µ*) (≥0.217 h^−1^), that was comparable to that of the wild-type (OD_630_ 1.53±0.004, and *µ* 0.259±0.008 h^−1^ respectively) (Table S1). However, two mutants with altered lag phases of 4.30±0.1 h (LUX1D2) and 0.35±0.087 h (LUX12E2) were excluded from selection. An additional mutant (LUX2A1), that failed to grow on a minimal growth medium, was excluded from selection as a putative auxotroph.

Preservative susceptibility of the 94 sub-group was determined by agar dilution method. In total, 76 of the 94 mutants demonstrated altered susceptibility to that of the wild-type (Table S1). Of these, 70 mutants demonstrated a two-fold decrease in the MIC of benzisothiazolinone (BIT), six mutants demonstrated a two-fold increase in MIC of methylisothiazolinone (MIT), while five mutants demonstrated a two-fold increase in MIC of dimethyl dimethylol hydantoin (DMDMH) (Table S1). None of the 94 mutants demonstrated altered susceptibility to phenoxyethanol (PH) or the chloromethylisothiazolinone and methylisothiazolinone blend (CMIT/MIT). Five mutants had altered susceptibility to both MIT and BIT (Table S1). Only one mutant (LUX1D2) demonstrated a gross change in preservative susceptibility with a greater than four-fold decrease in MIC of DMDMH and a two-fold decrease in MIC of BIT (Table 1); this mutant was also determined to have altered growth characteristics.

The top five mutants (Table 1), identified as suitable candidate reporters by the screening process, demonstrated comparable consistent levels of bioluminescence that increased in relation to increasing cell numbers over 48 h (data not shown). Based on the criteria for assessing performance, the *P. aeruginosa* LUX12H5 mutant was selected as a suitable reporter strain and used in subsequent evaluations. The long-term stability of the mini-Tn5 insertion within the LUX12H5 mutant was assessed over 6 weeks continuous culture in the absence of tetracycline, via enumeration on TSA plates with and without tetracycline. Levels of bioluminescence were consistent over time this time and the transposon insertion remained stable in the absence of antibiotic selection. Stable bioluminescence was required for application in PET and maintenance of the reporter as a laboratory strain.

**Fig. 1.**
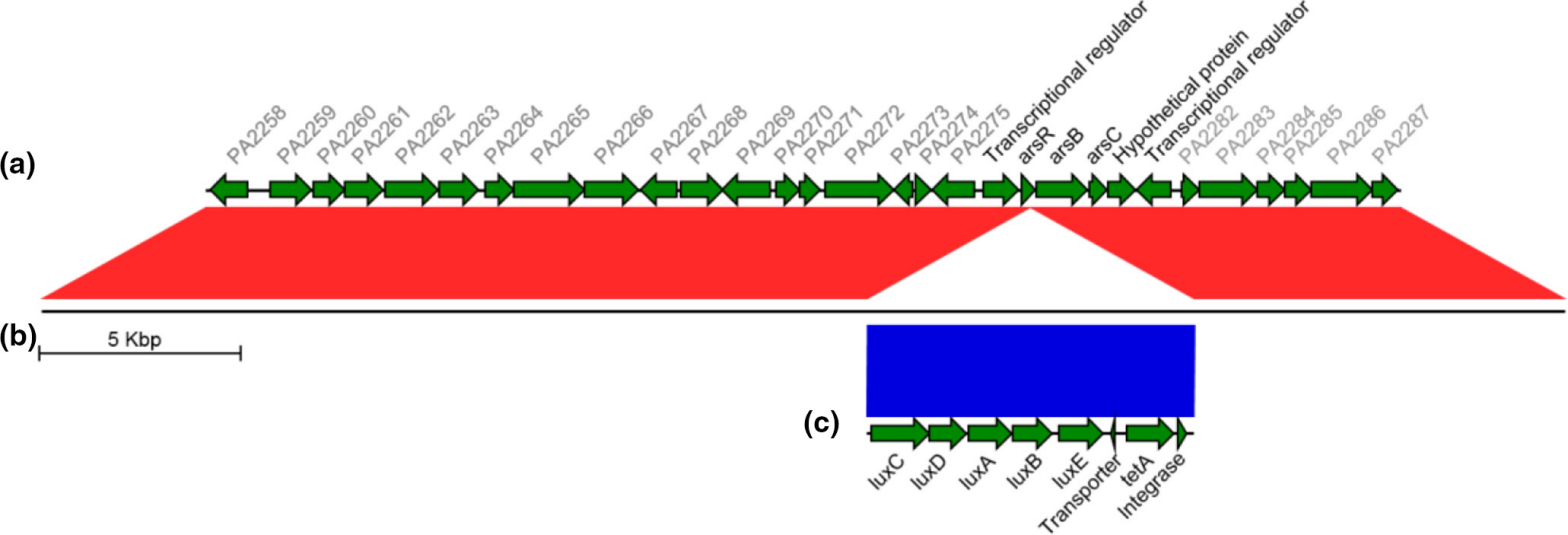
Genomic location of mini-Tn5-*luxCDABE* integration in the LUX12H5 mutant. The genomic region surrounding the mini-Tn5-*luxCDABE* in mutant LUX12H5 (**b**) is compared to the genome sequence of *P. aeruginosa PAO1* (**a**) and the sequence of the mini-Tn5-*LuxCDABE* (**c**). Regions of synteny are linked, with red indicating matches on the forward strand, and blue the reverse. Predicted gene products for PA2276 to PA2281 are marked.

Whole genome sequencing of the top five *P. aeruginosa* RW110 bioluminescence reporters (Table 1) was performed to map the insertion of the mini-Tn5 construct (Table 2). Insertion of the transposon reporter did not result in genomic rearrangements, and in each case the mini-Tn5 was confirmed to be 100 % identical in sequence to the donor construct using blast [32]. Two mutants, LUX12H5 and LUX12H8, showed a mini-Tn5 insertion within the same genomic region of *P. aeruginosa* RW110, disrupting a gene (PA2277) homologous to a predicted ArsR-like protein within the reference strain *P. aeruginosa* PAO1 (Table 2). Genomic examination of region surrounding the mini-Tn5-*luxCDABE* in LUX12H5 (Fig. 1) demonstrated it had inserted into a locus encoding genes predicted to function in arsenic resistance [33] and was flanked upstream and downstream by transcriptional regulator genes. This confirmed that the Tn5 insertion had not disrupted essential gene(s) in the reporter strain.

### Bioluminescence and viability correlates in the absence of antimicrobials

**Fig. 2.**
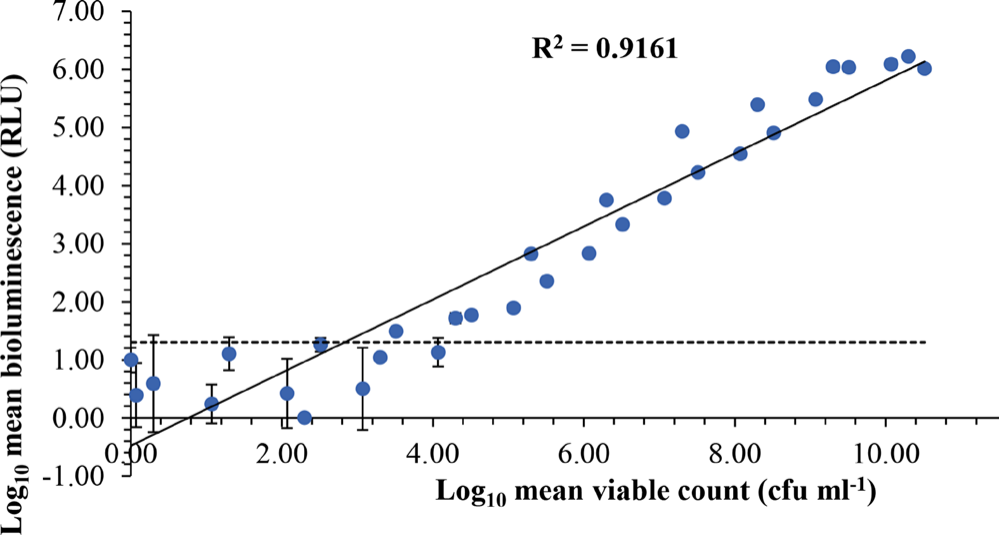
The direct correlation between light emission and viability observed for *P. aeruginosa* LUX12H5. A positive correlation between bioluminescence (relative light units [RLU]) and viable counts (c.f.u. ml^−1^) of *P. aeruginosa luxCDABE* mutant LUX12H5 cultured in TSB without preservative or antibiotic. Overnight cultures of the biosensor were pelleted by centrifugation and diluted in TSB. Data are shown as the Log_10_ transformed mean±standard deviation of duplicate experiments. The dotted line shows the threshold for detectable bioluminescence as ≥1.3 Log_10_ RLU. The assay demonstrated a range of six orders of magnitude with a minimum and maximum limit of detection as respectively 3.5–4 Log_10_ and >10 Log_10_ c.f.u. ml^−1^.

In the absence of antimicrobials, the reporter strain produced consistent levels of bioluminescence, and a positive correlation (*r^2^*=0.9161) was established between light output and viable counts (Fig. 2). The levels of bioluminescence were a reliable indicator of cell numbers in the experimental system and produced highly accurate light emission-viability correlates down to a minimum limit of detection (≥4 Log_10_ c.f.u. ml^−1^; Fig. 2). Between 4 and 10 Log_10_ c.f.u. ml^−1^ there was very limited variance in the light emission readings (Fig. 2). However, the reliability of enumerating cell number by bioluminescence greatly decreased after preservative and/or antibiotic exposure.

### Bioluminescence shows the early and transient effects of isothiazolinone preservatives on bacterial metabolism

**Fig. 3.**
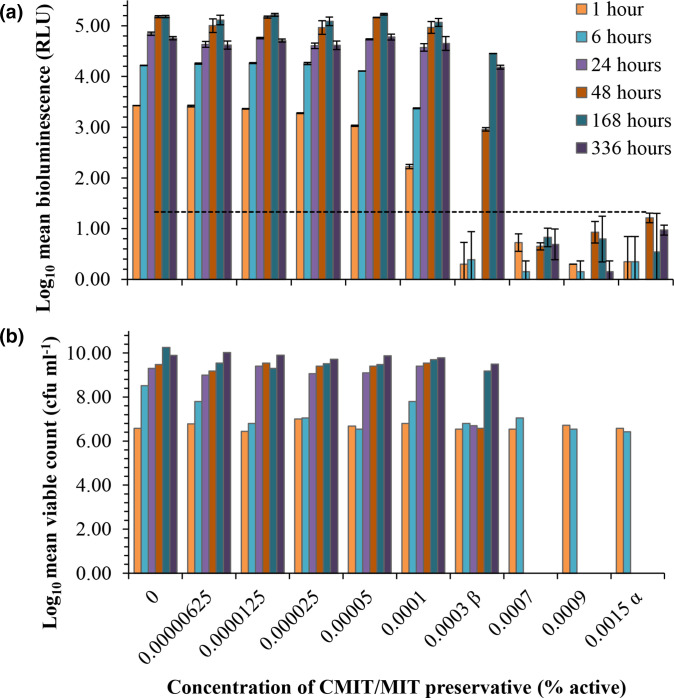
Rapid loss of *P. aeruginosa* LUX12H5 light emission but not viability in the presence of isothiazolinone preservatives. (**a**) Bioluminescence (relative light units [RLU]) and (**b**) viability (c.f.u. ml^−1^) of *P. aeruginosa luxCDABE* mutant LUX12H5 challenged with a range of CMIT/MIT preservative concentrations were monitored in parallel over a period of 2 weeks at time points of 1, 6, 24, 48, 168 and 336 h. Data are shown as the Log_10_ transformed mean±standard deviation of duplicate experiments. ^β^ MIC of CMIT/MIT was determined as 0.0007 % active. ^α^ The maximum EU regulated level of CMIT/MIT 0.0015 % active [29]. The dotted line shows the threshold for detectable bioluminescence as ≥1.3 Log RLU.

The *P. aeruginosa* LUX12H5 reporter was subsequently evaluated in a series of tests typical of PET. The effect of low to in-use levels of CMIT/MIT on the bioluminescence and viability of the reporter strain over a period of 2 weeks is shown in Fig. 3. After short exposure times of 1 and 6 h, bioluminescence decreased in relation to increasing preservative concentration: a 1.2 Log_10_ reduction in RLU from that of the control at 1 h (3.43 Log_10_ RLU) in the presence of sub-MIC 0.0001 % active CMIT/MIT (Fig. 3a), ranging up to a 3.28 Log_10_ reduction in RLU in the presence of 0.0015 % active CMIT/MIT (Fig. 3a). This early decrease in bioluminescence did not correlate to a reduction in viability for *P. aeruginosa* LUX12H5 (Fig. 3b), and the same relationship was observed in all ten of the highest performing mutants (listed in Tables 1 and S1). Interestingly, the reduction in bioluminescence was transient for concentrations of CMIT/MIT ≤0.0007 % active. After 6 h exposure to 0.0001 %, and 48 h exposure to 0.0003 % CMIT/MIT, bioluminescence was restored to that of the control at 1 h (c.f.u. ml^−1^ had altered by ≤1 Log_10_) (Fig. 3a). This suggested the recovery of the starting *P. aeruginosa* inoculum from metabolic inhibition by the sub-lethal preservative concentrations. This response to isothiazolines would not have been apparent by traditional plate count assay.

**Fig. 4.**
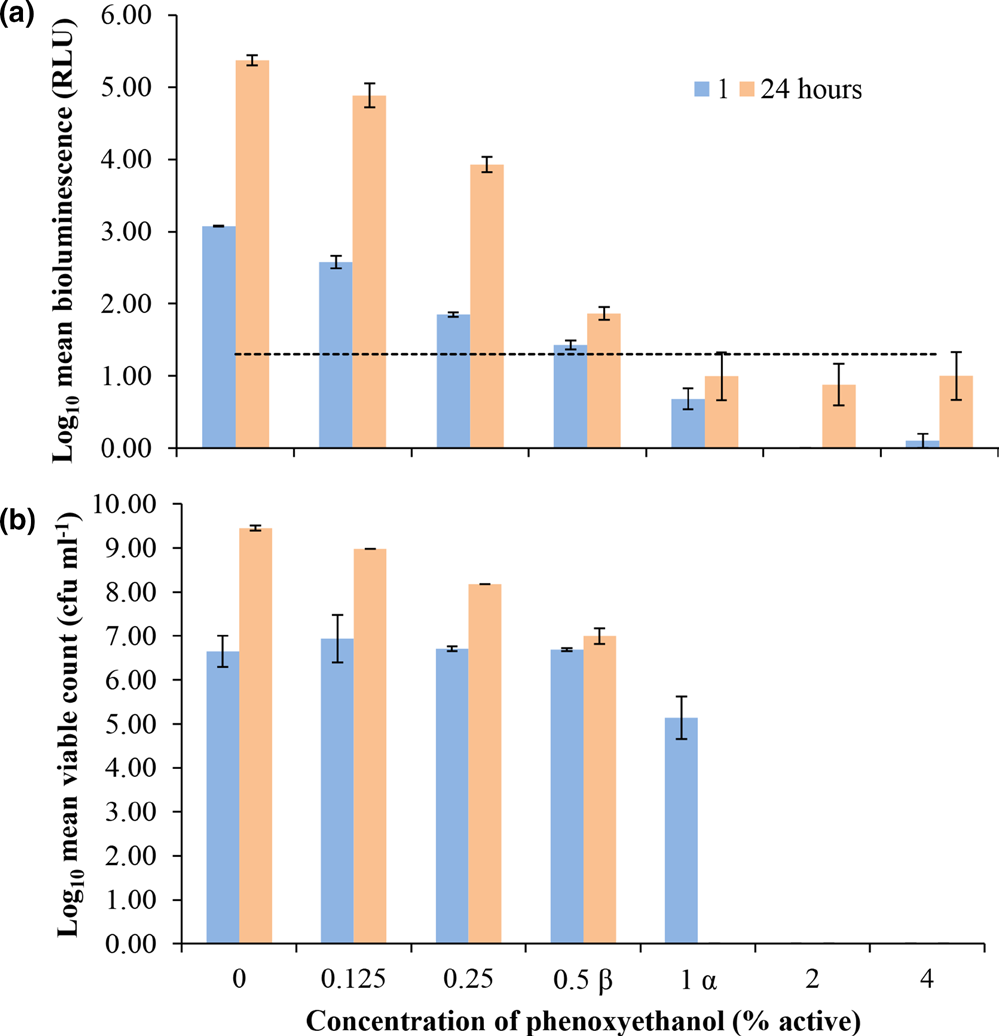
The recovery of *P. aeruginosa* LUX12H5 light emission in the presence of sub-lethal concentrations of phenoxyethanol preservatives. (**a**) Bioluminescence (relative light units [RLU]) and (**b**) viability (c.f.u. ml^−1^) of *P. aeruginosa luxCDABE* mutant LUX12H5 challenged with a range of phenoxyethanol (PH) preservative concentrations were monitored in parallel for 24 h. Data are shown as the Log_10_ transformed mean±standard deviation of duplicate experiments. ^α^ 1 % active, the maximum EU regulated level [29]. ^β^ The MIC of PH was determined as 0.5 % active. Greater than 24 h exposure time to 1 % PH achieved complete kill of Log 6 c.f.u. ml^−1^. The dotted line shows the threshold for detectable bioluminescence as ≥1.3 Log RLU.

A similar bioluminescence signal trend was also observed after 24 h exposure to sub-MIC of phenoxyethanol at 0.125 and 0.25 % v/v active: RLU decreased respectively by 0.5 and 1.23 Log_10_ from the control at 1 h (3.08 Log_10_ RLU), recovering to 4.89 and 3.93 Log_10_ RLU respectively at 24 h (Fig. 4a). However, metabolic inhibition persisted in the presence of 0.5 % v/v phenoxyethanol (MIC) with a mean of 6.99±0.26 Log_10_ c.f.u. ml^−1^ emitting a mean of 1.86±0.21 Log_10_ RLU (Fig. 4).

**Fig. 5.**
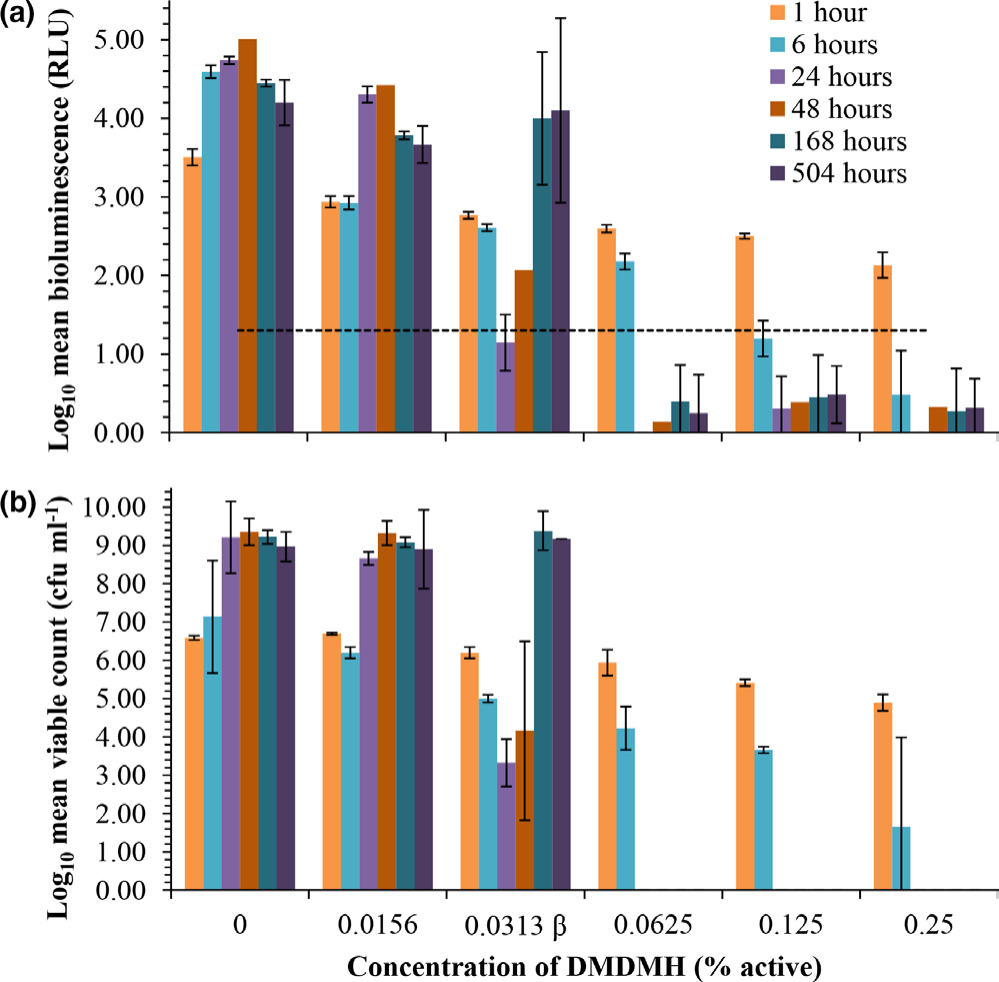
The metabolic recovery and proliferation of DMDMH injured cells. (**a**) Bioluminescence (relative light units [RLU]) and (**b**) viability (c.f.u. ml^−1^) of *P. aeruginosa luxCDABE* mutant LUX*12H5* challenged with a range of DMDMH preservative concentrations at 0–0.25 % active, were monitored in parallel over a period of 3 weeks. Data are shown as the Log_10_ transformed mean±standard deviation of duplicate biological experiments. Survivors had fully recovered exposure to 0.0313 % DMDMH by 168 h. ^β^ MIC of DMDMH determined as 0.0313 % active. The maximum EU regulated level is 0.6 % active [29]. The dotted line shows the threshold for detectable bioluminescence as ≥1.3 Log RLU.

In contrast, bioluminescence and viability measurements demonstrated the recovery of surviving cells exposed to the MIC of DMDMH (Fig. 5). After 24 h exposure to 0.0313 % DMDMH decreased RLU by 2.36 Log_10_, and c.f.u. ml^−1^ by 3.26 Log_10_, from that of the control at 1 h (3.51 Log_10_ RLU and 6.59 c.f.u. ml^−1^) (Fig. 5). This rendered bioluminescence to undetectable levels, and the number of viable cells (3.32±0.6 Log_10_ c.f.u. ml^−1^) close to the lower detection limits of the traditional agar plate count assay (~1×10^3^ c.f.u. ml^−1^) [17]. However, after 1 week the surviving cells cultured in the presence of 0.0313 % DMDMH had proliferated: levels of bioluminescence and viability were restored and equivalent to that of the control at 168 h (Fig. 5). In this capacity, the assay could provide manufacturers with a means to identify, and consequently avoid, conditions that facilitate the rebound growth of spoilage bacteria. Due to the efficacy of the CMIT/MIT preservative in the experimental system there was a limited range in the number of recovered surviving cells: the starting inoculum was either completely killed or increased from 6.42 to 10.02 Log_10_ c.f.u. ml^−1^ after a period of inhibition, over the weeks of incubation. However, it was apparent that in a system containing preservative, the number of viable cells could not be estimated based on the levels of bioluminescence, as the RLU of preservative exposed cells could be lower than that of equivalent numbers cultured in the absence of preservative (Fig. 3). The discrepancy between bioluminescence and viable cells was particularly problematic during early measurements. For example, 6.57 Log_10_ c.f.u. ml^−1^ exposed to 0.0003 % CMIT/MIT for 48 h produced 2.96±0.035 Log_10_ RLU, whereas equivalent numbers of the control produced 3.43±0.002 Log_10_ RLU (Fig. 3). In addition, the sustained absence of detectable bioluminescence was not a reliable indicator of complete kill. For example, inhibitory but sub-lethal concentrations of CMIT/MIT could reduce the light output of the reporter strain to undetectable levels, and the minimum limit of the assay prevented the accurate detection of low numbers of surviving cells.

### Correlation of bioluminescence and viable counts after a ciprofloxacin challenge

**Fig. 6.**
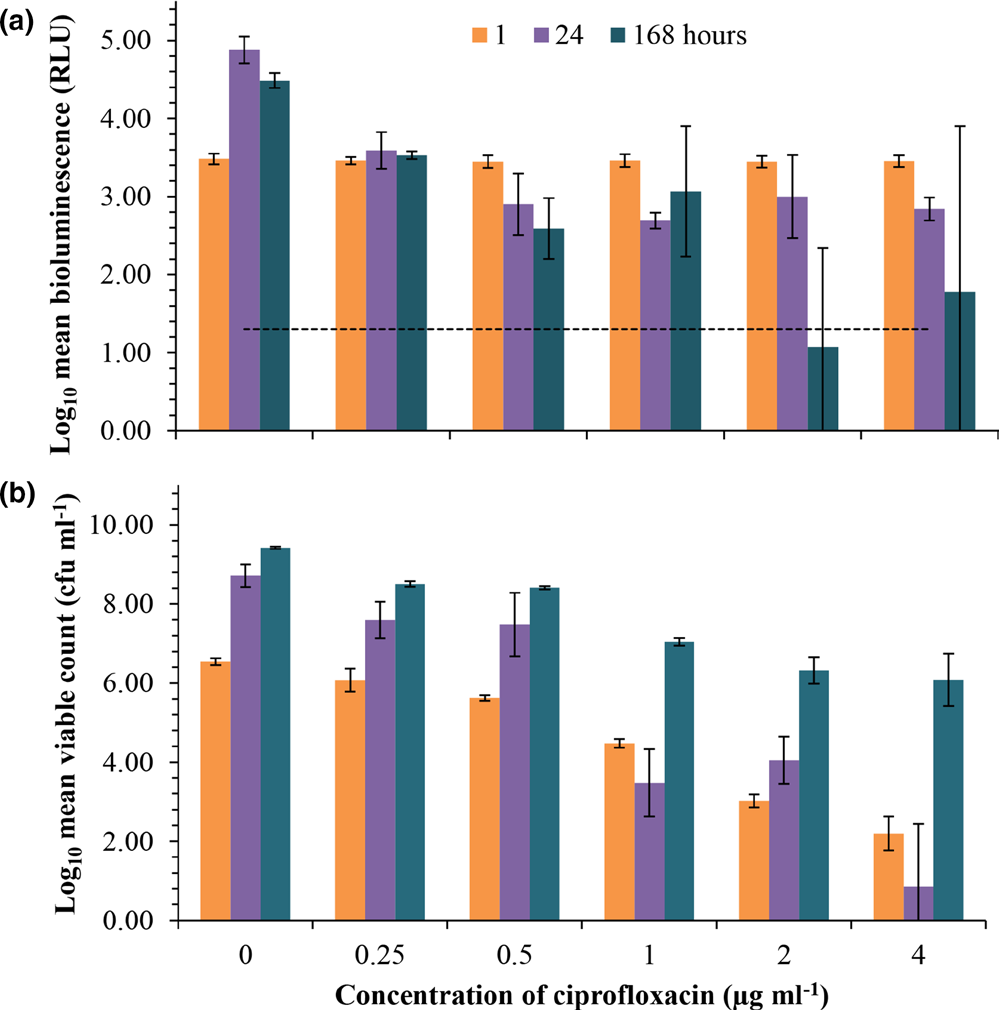
Reduced viable counts of *P. aeruginosa* LUX12H5 but persisting bioluminescence in the presence of ciprofloxacin. (**a**) Bioluminescence (relative light units [RLU]) and (**b**) viability (c.f.u. ml^−1^) of *P. aeruginosa luxCDABE* mutant lux12H5 challenged with a range of ciprofloxacin antibiotic concentrations were monitored in parallel over a period of 7 days at timepoints of 1, 24, and 168 h. Data are shown as the Log_10_ transformed mean±standard deviation of duplicate experiments. Data for concentrations of 0.01625 and 0.125 µg ml^−1^ not shown. The dotted line shows the threshold for detectable bioluminescence as ≥1.3 Log_10_ RLU.

The reduction in bioluminescence but not viability during short-term (<6 h) isothiazolinone preservative exposure suggested that the *P. aeruginosa lux* reporter may reveal the transient effects of an antimicrobial on metabolism. A ciprofloxacin challenge was used to explore whether converse correlations between RLU and viability were observed for an antimicrobial that specifically targets DNA replication as opposed to metabolism (Fig. 6). The *P. aeruginosa* LUX12H5 ciprofloxacin MIC was 0.5 µg ml^−1^, as determined by agar dilution [31]. Light emission at a range of concentrations up to this inhibitory level of antibiotic were evaluated (Fig. 6). In contrast to preservative challenges, the overall decline in viable cell counts (≤6.54 Log_10_ c.f.u. ml^−1^, Fig. 6b) was greater than that of the decline in bioluminescence (≤2.4 Log_10_ RLU, Fig. 6a) following a ciprofloxacin challenge; cells were metabolically active but not able to proliferate. This suggests that when analysed in combination, the relationship between bioluminescence and viable counts of the reporter strain can demonstrate an antimicrobial effect on DNA replication or metabolism.

## Discussion

There is a need for rapid PET methods to keep pace with regulatory and public demands for milder preservative systems with less impact on the environment. Rapid PET technologies that bring microbial viability assays from their current retrospective microbial growth status into real-time monitoring, will facilitate the high throughput testing required to meet these demands. Here we uniquely explore the utility of bioluminescence as a measure for bacterial viability within PET, and as such it was conducted in a simple liquid system with a limited number of preservatives. The further impact of a complex formulation that may contain detergents and surfactants, on bacterial metabolism and consequently bioluminescence was not evaluated.

### Successful isolation of a stable, chromosomally encoded *P. aeruginosa lux* reporter

We uniquely used random mini-Tn5-*lux* mutagenesis to create bespoke bioluminescent mutants of a preservative tolerant *P. aeruginosa* industrial strain suitable for use in PET. A promoter-less mini-Tn5 derivative carrying the *luxCDABE* operon was used to select for transcribed *lux* gene fusions [19] in mutants, with a range of bioluminescent capabilities. The approach also worked as promoter-probe screen, identifying constitutive promoters for use in future *P. aeruginosa* reporter constructs. Overall, the approach was successful in producing a stable light-emitting biosensor, within a relevant industrial *P. aeruginosa* strain background, for evaluation in PET. Despite the fact transposon mutagenesis can be used to isolate mutants of disrupted phenotype, few of the mutants within the phenotype evaluated sub-group of 94 (Table S1) demonstrated altered growth characteristics or auxotrophy. The study also demonstrated that mini-Tn5 insertions within *P. aeruginosa* can be highly stable, with the selected LUX12H5 reporter remaining fully functional over weeks of growth passage in the absence of the transposon-selecting antibiotic. The stability of the selected reporter strain meant its maintenance would not differ from that of other challenge test bacteria. To avoid phenotypic change, the Pharmacopoeia compendia recommend that test organisms are lyophilised or cryogenically stored, and the number of passages from the main stock culture is kept to a minimum with a limit of five. Based on the stability findings of this study, the number of passages for the LUX12H5 reporter should be limited to three.

The majority (77 of 94) of the sub-group demonstrated altered susceptibility to preservatives, with a two-fold alteration in the MIC (Table S1). However, this change was in line with natural variations observed within a culture for this type of phenotypic testing [34]. In contrast, Shah and Naseby [35] reported a drastic increase in susceptibility to benzalkonium chloride (eight-fold reduction in MIC) for a transformed *P. aeruginosa* with a plasmid borne *Lux* cassette under a constitutive promoter. Large alterations in preservative MIC or growth rate were not observed with the *P. aeruginosa* LUX12H5 reporter, suggesting that the chromosomal integration of *lux* genes may help reduce the metabolic burden of the bioluminescent construct. Only one of the evaluated knockout mutations, LUX1D2, resulted in altered susceptibility to unrelated preservatives BIT and DMDH. In total, 36 of 94 mutants subjected to screening fulfilled the selection criteria for application to PET, indicating that the phenotype and bioluminescent stability of the reporter constructs was a consistent trait that could be identified within the overall mutant bank. Using the same approach to construct bespoke reporters in other Gram-negatives such as *Burkholderia* and Enterobacteriaceae with industrial relevance [16] should therefore be feasible.

### Applicability of bioluminescence to PET and understanding preservative antimicrobial mechanisms of action

A key requirement for the application of bioluminescence in PET was to establish correlation between light output and viable counts. As acceptance criteria of PET stipulate a reduction in the viability of test organisms, not just the inhibition of growth/metabolism. A strict correlation was established between bioluminescence and viable counts in the absence of preservative (Fig. 2). This finding was consistent with those previously reported in the validation study of bioluminescent *P. aeruginosa* constructs*,* constitutively expressing plasmid borne *lux* genes, as a rapid microbiological quantification tool [13]. Although the range of the bioluminescent assay was impressive at six orders of magnitude, the sensitivity or minimum limit of detection was disappointing at around 4 Log_10_ c.f.u. ml^−1^. This level of sensitivity failed to comply with published PET acceptance criteria that generally require confirmation of a 99 % kill or 4 Log_10_ reductions in the test bacteria over time. Shah and Naseby [13] reported better sensitivity with a lower detection limit of 10^3^ c.f.u. ml^−1^, but this difference may have resulted from technical protocol differences such as the luminometer used, sample volume measured or the point at which they elected to assign their lower limit. Our high-throughput and small volume 96-well system, which was suited to PET, was capable of detecting light signals at 10^3^ c.f.u. ml^−1^ (Fig. 2) but this was deemed to be below a reproducible and accurate threshold. Separating the *luxCDABE* genes into two smaller functional units, *luxAB* and *luxCDE*, may provide a means of improving the performance of the reporter strain, as it has been shown to improve the efficiency of transcription and/or translation of the *lux* system in other bacteria [36]. However, high expression of luciferase systems has been shown to impaired growth characteristics [6] and may thereby reduce the suitability of the reporter strain for PET.

In this study we evaluated the performance of the *P. aeruginosa* LUX12H5 bioluminescent reporter in presence of three classes of preservative: multiple isothiazolinones, phenoxyethanol and the formaldehyde-releaser DMDMH. Previous studies, examining single agents such as benzalkonium chloride against bioluminescent *P. aeruginosa.* [35] reported no significant difference between bioluminescence and enumeration by plate count. Benzalkonium chloride predominantly targets the cell wall and membrane in bacteria [37], a mode of action that is unlikely to directly interfere with luminescence. In contrast, isothiazolinone and DMDMH agents are known to have an intracellular mode of action that can impede metabolism and therefore bioluminescence. Although we found that bioluminescence and viable counts failed to correlate after short-term exposures to isothiazolinone preservatives, over the long-term duration of the efficacy test the light output to viability correlation improved. In PET, it is important to evaluate short-term and long-term efficacy with the duration of a full test being 28 days. We also found that cells surviving prolonged exposure to low-levels of preservatives could emit less bioluminescence than cells cultured in the absence of preservative. This made the quantification of the surviving test bacteria by bioluminescence levels alone unreliable.

Discrepancies between the bioluminescence and viable counts of reporter strains have been attributed to, and utilised in resolving, the antimicrobial mechanism of action [10, 38]. In bacteria, isothiazolinones are thought to react chemically with thiol containing cytoplasmic and membrane bound enzymes [39]. The rapid decline in bioluminescence but not viability would suggest that isothiazolinones have an immediate effect on bacterial metabolism and/or specifically energy generating metabolic processes, as bioluminescence is dependent on the reducing power of FMNH_2_ and a functioning intracellular biochemistry. The transient effect of sub-lethal concentrations of isothiazolinone on bioluminescence inhibition is not fully understood, it is possible that the preservative was being removed from the cells via efflux. The utility of the bioluminescent reporter strain for discriminating the effects of an antimicrobial on multiplication or metabolism was confirmed following a ciprofloxacin challenge. Exposure to ciprofloxacin, which specifically targets DNA gyrase, resulted in a reversion of the correlation observed for isothiazolinone preservatives: viable counts declined before levels of bioluminescence. This finding was consistent with those previously reported for ciprofloxacin challenged self-bioluminescent *P. aeruginosa* biofilms [40]. An important limitation of the current research into the applicability of whole-cell bioluminescence to PET is the need to consider the effects of antimicrobial agents/concentrations with a bacteriostatic action, capable of inhibited cellular metabolism (and consequently light emission) and growth but that do not result in cell death.

### Bioluminescence reveals the rebound growth of metabolically active cells after DMDMH exposure

Bacterial rebound growth in product released to trade, also known as the phoenix phenomenon [17], can be extremely costly to manufacturers. The situation is characterised by a decline in viable cell numbers before growth resumes, and can generally be attributed to inadequacies in the preservative system [17]. This study highlighted one of the challenges to formulation scientists. The analysis of bioluminescence and viable counts revealed that DMDMH has a rapid and multifactorial effect on both bacterial metabolism and viability. Even low-levels of DMDMH initially reduced viability to the lower limits of detection by conventional agar plate assays and could therefore wrongly be considered as adequate protection against spoilage over the long-term. However, bioluminescence indicated the metabolic recovery/activity of a small group of cells that had survived DMDMH preservative exposure and were able to proliferate. The DMDMH susceptibility of the surviving population was not reassessed in this study, but previous findings by Rushton *et al.* [21] suggest that the development/selection of DMDMH resistant organisms in industry is a concern that reinforces the importance of thorough PET.

In summary, random mini-Tn5-*lux* mutagenesis combined with the systematic screening of the mutant library successfully constructed and identified a bioluminescent phenotype suitable for application in PET. A strong correlation between light output and viable counts was established in the absence of preservative. However, due to the multi-factorial nature of the evaluated preservatives, and their rapid putative effect on bacterial metabolism, the desired correlation during testing was not reliably observed. In this capacity, lux reporters fall short due to their dependency on active metabolism. Consequently, this methodology would not be suitable as an alternative to culture-based PET. The relationship between light and viability was indicative of the antimicrobial mechanism of action. In this respect, lux reporters can be used to advance the understanding of antimicrobial mechanisms of action. Demonstrating the multi-factorial nature of CMIT/MIT and DMDMH in disrupting metabolic processes in addition to the characterised interactions they have respectively with thiol groups, or the amide and amino groups of protein molecules [39]. *P. aeruginosa* LUX12H5 bioluminescent reporter demonstrated the propensity for cells exposed to sub-inhibitory concentrations of DMDMH to recover metabolic activity and actively proliferate as rebound growth.

## Supplementary Data

Supplementary material 1
