# Supplementary material for: Construction and evaluation of a bioluminescent Pseudomonas aeruginosa reporter for use in preservative efficacy testing
Source: Microbiology (Reading). 2021 Aug 12;167(8):001072. doi: 10.1099/mic.0.001072 (PMC8513615; doi:10.1099/mic.0.001072)
Supplement: Supplementary material 1 [file mic-167-1072-s001.pdf]

Construction and evaluation of a bioluminescent *Pseudomonas aeruginosa* reporter for use in preservative efficacy testing

Laura Rushton [a#], Denise Donoghue [b], Matthew Bull [a], Peter Jay [b] and Eshwar Mahenthiralingam [a#]

[a]Cardiff School of Biosciences, Cardiff University, Cardiff, UK

[b]Unilever R&D Port Sunlight, Wirral, Merseyside, UK

#Correspondence to: MahenthiralingamE@cardiff.ac.uk

#Co-correspondence to: Dr. Laura Rushton, RushtonL3@cardiff.ac.uk

<u>Contents of Supplemental Materials</u>

1. **Supplemental Figure S1.** 89 of the 94 *Pseudomonas aeruginosa luxCDABE* mutants characterised as a sub-group

.

Supplemental Table S1. 89 of the 94 *Pseudomonas aeruginosa luxCDABE* mutants characterised as a sub-group.

mutant ID	Bioluminescence/ OD_{630}		Growth characteristics						Preservative MIC (% active)				
	Revival	Sub-culture	Growth rate μ (±STDEV)		Lag phase hours (±STDEV)		Maximum growth OD (±STDEV)		MIT	CMIT/ MIT	BIT	PH	DMH
**Wild-type**	0	0	0.259	(±0.008)	1.106	(±0.104)	1.525	(±0.004)	0.00468	0.0025	0.01875	0.5	0.0625
**1E5**	500524	307781	0.251	(±0.004)	0.922	(±0.057)	1.613	(±0.002)	0.00468	0.0025	0.01875	0.5	0.0625
**1C2**	558231	303020	0.230	(±0.004)	0.447	(±0.053)	1.631	(±0.002)	0.00468	0.0025	0.01875	0.5	0.125
**1A5**	613360	279718	0.267	(±0.007)	0.817	(±0.094)	1.574	(±0.004)	0.00468	0.0025	0.009375	0.5	0.0625
**1E4**	723179	278051	0.261	(±0.007)	1.184	(±0.089)	1.573	(±0.003)	0.00468	0.0025	0.01875	0.5	0.0625
**1G2**	652688	276070	0.253	(±0.005)	1.180	(±0.077)	1.564	(±0.003)	0.00468	0.0025	0.009375	0.5	0.0625
**1E10**	537943	270331	0.246	(±0.006)	0.543	(±0.084)	1.582	(±0.003)	0.00468	0.0025	0.01875	0.5	0.125
**1F7**	524933	234079	0.254	(±0.006)	1.016	(±0.088)	1.551	(±0.003)	0.00468	0.0025	0.009375	0.5	0.0625
**3H6**	233692	230867	0.235	(±0.003)	0.996	(±0.050)	1.608	(±0.002)	0.00468	0.0025	0.009375	0.5	0.0625
**12G2**	108598	226003	0.253	(±0.007)	1.031	(±0.097)	1.545	(±0.004)	0.00468	0.0025	0.009375	0.5	0.0625
**1F6**	265018	209758	0.233	(±0.003)	1.101	(±0.053)	1.613	(±0.002)	0.00468	0.0025	0.009375	0.5	0.0625
**12F7**	215970	190575	0.232	(±0.005)	0.835	(±0.077)	1.619	(±0.003)	0.00468	0.0025	0.009375	0.5	0.0625
**3G11**	361941	188951	0.233	(±0.003)	1.152	(±0.046)	1.608	(±0.002)	0.00468	0.0025	0.009375	0.5	0.0625
**12D4**	336738	186593	0.229	(±0.006)	1.210	(±0.101)	1.537	(±0.004)	0.00468	0.0025	0.009375	0.5	0.0625
**1H9**	336046	185519	0.233	(±0.003)	0.770	(±0.047)	1.616	(±0.002)	0.00468	0.0025	0.01875	0.5	0.0625
**5H4**	228093	168866	0.231	(±0.003)	0.961	(±0.051)	1.594	(±0.002)	0.00468	0.0025	0.01875	0.5	0.0625

**12G12**	161446	160127	0.290	(±0.008)	1.261	(±0.091)	1.579	(±0.004)	0.00468	0.0025	0.01875	0.5	0.0625
**8D8**	358655	156932	0.249	(±0.004)	0.989	(±0.051)	1.608	(±0.002)	0.00468	0.0025	0.009375	0.5	0.0625
**7F4**	381744	154414	0.249	(±0.004)	0.796	(±0.053)	1.635	(±0.002)	0.00468	0.0025	0.01875	0.5	0.125
**4H11**	263657	141951	0.220	(±0.003)	0.665	(±0.048)	1.601	(±0.002)	0.00468	0.0025	0.01875	0.5	0.125
**7F8**	394414	140952	0.271	(±0.006)	0.995	(±0.070)	1.587	(±0.003)	0.00468	0.0025	0.009375	0.5	0.0625
**8F4**	389492	123420	0.275	(±0.006)	1.164	(±0.076)	1.565	(±0.003)	0.00468	0.0025	0.01875	0.5	0.125
**12F12**	269610	123116	0.231	(±0.006)	0.566	(±0.105)	1.596	(±0.004)	0.00468	0.0025	0.01875	0.5	0.0625
**3G12**	213908	107187	0.217	(±0.003)	0.870	(±0.046)	1.604	(±0.002)	0.00468	0.0025	0.009375	0.5	0.0625
**7F1**	296741	102566	0.260	(±0.005)	0.894	(±0.066)	1.600	(±0.003)	0.00468	0.0025	0.01875	0.5	0.0625
**12E2** [1]	198798	102515	0.233	(±0.005)	0.350	(±0.087)	1.618	(±0.003)	0.00468	0.0025	0.009375	0.5	0.0625
**9D1**	319237	100084	0.284	(±0.009)	1.214	(±0.093)	1.518	(±0.004)	0.00468	0.0025	0.009375	0.5	0.0625
**5G3**	460199	86325	0.232	(±0.004)	0.942	(±0.060)	1.589	(±0.002)	0.00468	0.0025	0.009375	0.5	0.0625
**3B7**	13578	71498	0.246	(±0.004)	1.039	(±0.059)	1.576	(±0.002)	0.00468	0.0025	0.009375	0.5	0.0625
**4H3**	145198	67497	0.274	(±0.005)	0.902	(±0.062)	1.599	(±0.003)	0.00468	0.0025	0.009375	0.5	0.0625
**8D9**	97417	66168	0.245	(±0.003)	0.667	(±0.050)	1.637	(±0.002)	0.00468	0.0025	0.009375	0.5	0.0625
**12E1**	164057	65981	0.241	(±0.005)	0.846	(±0.084)	1.620	(±0.003)	0.00468	0.0025	0.009375	0.5	0.0625
**12G7**	227894	54498	0.236	(±0.006)	0.843	(±0.093)	1.564	(±0.003)	0.00468	0.0025	0.009375	0.5	0.0625
**12E8**	368671	38182	0.230	(±0.005)	0.955	(±0.090)	1.592	(±0.003)	0.00468	0.0025	0.009375	0.5	0.0625
**1B10**	509373	31540	0.253	(±0.007)	1.439	(±0.098)	1.535	(±0.004)	0.00468	0.0025	0.009375	0.5	0.0625
**6H8**	474182	28472	0.225	(±0.004)	0.887	(±0.066)	1.611	(±0.003)	0.00468	0.0025	0.009375	0.5	0.0625
**2A1** [1]	18404	28472	0.258	(±0.006)	1.300	(±0.777)	1.554	(±0.003)	0.00468	0.0025	0.01875	0.5	0.0625
**12A6**	198890	22190	0.267	(±0.008)	0.716	(±0.102)	1.568	(±0.004)	0.00468	0.0025	0.009375	0.5	0.0625
**11F6**	233106	22006	0.264	(±0.008)	1.453	(±0.096)	1.521	(±0.004)	0.00468	0.0025	0.009375	0.5	0.0625

**11E7**	31761	21328	0.279	(±0.009)	1.126	(±0.100)	1.552	(±0.004)	0.00936	0.0025	0.009375	0.5	0.0625
**12G8**	394608	18516	0.227	(±0.005)	1.139	(±0.085)	1.597	(±0.003)	0.00468	0.0025	0.009375	0.5	0.0625
**12H12**	166429	17513	0.244	(±0.005)	0.768	(±0.081)	1.626	(±0.003)	0.00468	0.0025	0.009375	0.5	0.0625
**11G8**	249878	15338	0.265	(±0.008)	1.096	(±0.095)	1.549	(±0.004)	0.00468	0.0025	0.009375	0.5	0.0625
**7C7**	73946	14642	0.246	(±0.004)	0.617	(±0.063)	1.628	(±0.003)	0.00468	0.0025	0.009375	0.5	0.0625
**12D9**	401301	11812	0.258	(±0.006)	0.660	(±0.084)	1.626	(±0.003)	0.00468	0.0025	0.009375	0.5	0.0625
**5G1**	1972	11584	0.241	(±0.003)	0.767	(±0.047)	1.644	(±0.002)	0.00468	0.0025	0.009375	0.5	0.0625
**8H8**	207559	10217	0.243	(±0.005)	0.850	(±0.070)	1.593	(±0.003)	0.00468	0.0025	0.01875	0.5	0.0625
**12F6**	191822	9922	0.270	(±0.008)	0.963	(±0.100)	1.560	(±0.004)	0.00468	0.0025	0.009375	0.5	0.0625
**5D5**	159274	7216	0.242	(±0.003)	0.958	(±0.045)	1.638	(±0.002)	0.00468	0.0025	0.009375	0.5	0.0625
**12G6**	75652	7033	0.245	(±0.006)	0.899	(±0.078)	1.613	(±0.003)	0.00468	0.0025	0.009375	0.5	0.0625
**12F3**	130209	6905	0.245	(±0.004)	0.517	(±0.063)	1.651	(±0.003)	0.00468	0.0025	0.009375	0.5	0.0625
**12F8**	263891	5756	0.225	(±0.005)	1.806	(±0.088)	1.590	(±0.003)	0.00468	0.0025	0.009375	0.5	0.0625
**11G9**	239493	5745	0.254	(±0.005)	0.930	(±0.076)	1.593	(±0.003)	0.00468	0.0025	0.009375	0.5	0.0625
**12F5**	196767	5323	0.242	(±0.006)	0.703	(±0.089)	1.584	(±0.003)	0.00468	0.0025	0.01875	0.5	0.0625
**12H6**	164419	4674	0.230	(±0.005)	0.755	(±0.088)	1.603	(±0.003)	0.00468	0.0025	0.009375	0.5	0.0625
**12D10**	14157	3831	0.287	(±0.009)	1.126	(±0.101)	1.589	(±0.004)	0.00468	0.0025	0.009375	0.5	0.0625
**12E9**	14978	3611	0.242	(±0.006)	0.673	(±0.085)	1.612	(±0.004)	0.00468	0.0025	0.009375	0.5	0.0625
**12G1**	88559	2978	0.238	(±0.005)	0.596	(±0.080)	1.631	(±0.003)	0.00468	0.0025	0.009375	0.5	0.0625
**12G9**	246997	2424	0.253	(±0.007)	0.674	(±0.106)	1.595	(±0.004)	0.00468	0.0025	0.009375	0.5	0.0625
**12H9**	314525	2215	0.237	(±0.005)	0.956	(±0.084)	1.609	(±0.003)	0.00468	0.0025	0.009375	0.5	0.0625
**12E10**	226721	1859	0.249	(±0.004)	1.229	(±0.064)	1.605	(±0.003)	0.00468	0.0025	0.009375	0.5	0.0625
**5G6**	539164	1733	0.229	(±0.003)	0.956	(±0.058)	1.639	(±0.002)	0.00468	0.0025	0.01875	0.5	0.0625

**12D3**	32600	1709	0.262	(±0.006)	0.954	(±0.074)	1.625	(±0.003)	0.00468	0.0025	0.01875	0.5	0.0625
**11E11**	152531	1528	0.273	(±0.008)	1.098	(±0.092)	1.537	(±0.004)	0.00468	0.0025	0.009375	0.5	0.0625
**12H1**	21638	1384	0.281	(±0.008)	0.921	(±0.094)	1.577	(±0.004)	0.00468	0.0025	0.009375	0.5	0.0625
**11C12**	54384	1260	0.272	(±0.007)	1.036	(±0.090)	1.587	(±0.004)	0.00936	0.0025	0.01875	0.5	0.0625
**11C5**	244648	990	0.245	(±0.006)	0.973	(±0.093)	1.597	(±0.004)	0.00468	0.0025	0.009375	0.5	0.0625
**11H5**	84538	476	0.279	(±0.008)	1.105	(±0.095)	1.545	(±0.004)	0.00468	0.0025	0.009375	0.5	0.0625
**5G9**	35656	432	0.247	(±0.004)	1.009	(±0.051)	1.628	(±0.002)	0.00468	0.0025	0.009375	0.5	0.0625
**11H7**	45531	304	0.269	(±0.007)	0.579	(±0.081)	1.621	(±0.003	0.00936	0.0025	0.009375	0.5	0.0625
**11H6**	69480	253	0.278	(±0.005)	0.883	(±0.062)	1.618	(±0.003)	0.00468	0.0025	0.009375	0.5	0.0625
**12C2**	181420	249	0.274	(±0.008)	0.810	(±0.096)	1.576	(±0.004)	0.00468	0.0025	0.01875	0.5	0.0625
**12E5**	14936	222	0.248	(±0.007)	0.641	(±0.092)	1.602	(±0.004)	0.00468	0.0025	0.01875	0.5	0.0625
**11F7**	96945	161	0.292	(±0.009)	1.242	(±0.096)	1.559	(±0.004)	0.00468	0.0025	0.009375	0.5	0.0625
**11B10**	141696	131	0.253	(±0.007)	1.439	(±0.098)	1.535	(±0.004)	0.00468	0.0025	0.009375	0.5	0.0625
**7H6**	433372	118	0.258	(±0.007)	1.450	(±0.101)	1.522	(±0.003)	0.00936	0.0025	0.009375	0.5	0.0625
**11G6**	22735	116	0.267	(±0.006)	0.960	(±0.077)	1.358	(±0.003)	0.00468	0.0025	0.009375	0.5	0.0625
**12H2**	32269	77	0.244	(±0.006)	0.715	(±0.090)	1.602	(±0.004)	0.00468	0.0025	0.009375	0.5	0.0625
**4F11**	841	69	0.254	(±0.005)	1.168	(±0.070)	1.563	(±0.003)	0.00468	0.0025	0.01875	0.5	0.0625
**11F9**	56084	56	0.272	(±0.008)	0.781	(±0.095)	1.570	(±0.004)	0.00468	0.0025	0.009375	0.5	0.0625
**11H11**	18027	54	0.255	(±0.005)	0.561	(±0.062)	1.629	(±0.003)	0.00468	0.0025	0.009375	0.5	0.0625
**11A10**	14049	53	0.267	(±0.007)	0.850	(±0.093)	1.591	(±0.004)	0.00468	0.0025	0.009375	0.5	0.0625
**7B7**	24645	45	0.270	(±0.006)	0.972	(±0.073)	1.593	(±0.003)	0.00468	0.0025	0.01875	0.5	0.0625
**11E9**	34096	43	0.279	(±0.008)	1.171	(±0.092)	1.557	(±0.004)	0.00468	0.0025	0.009375	0.5	0.0625
**11H8**	7233	40	0.274	(±0.006)	0.769	(±0.065)	1.619	(±0.003)	0.00468	0.0025	0.009375	0.5	0.0625

**11D11**	44115	39	0.282	(±0.009)	1.029	(±0.099)	1.520	(±0.004)	0.00468	0.0025	0.009375	0.5	0.0625
**12G11**	133760	38	0.260	(±0.007)	0.918	(±0.093)	1.575	(±0.004)	0.00468	0.0025	0.009375	0.5	0.0625
**9F12**	420	34	0.274	(±0.006)	0.927	(±0.077)	1.568	(±0.003)	0.00936	0.0025	0.009375	0.5	0.0625
**12G10**	26000	32	0.255	(±0.006)	0.704	(±0.083)	1.362	(±0.004)	0.00468	0.0025	0.009375	0.5	0.0625
**12H10**	12561	27	0.278	(±0.008)	0.841	(±0.099)	1.574	(±0.004)	0.00936	0.0025	0.009375	0.5	0.0625

Mutants ranked in order of bioluminescence/optical density after three sub-cultures in the absence of tetracycline selection.

Bioluminescence/optical density were calculated after: the ‘Revival’ of frozen stock cultures cultured for 24 hours in TSB with tetracycline; and ‘Sub-culture’, where revived strains were sub-cultured three times (24 hour cultures) in TSB without tetracycline. Bioluminescence measured as relative light units (RLU). Abbreviations: OD, optical density at 630 nm; MIC, minimum inhibitory concentration; MIT, methylisothiazolinone; CMIT/MIT, chloromethylisothiazolinone & methylisothiazolinone blend; BIT, benzisothiazolinone; PH, phenoxyethanol; DMDMDH, dimethyl dimethylol hydantoin; [1] Mutant excluded as it failed to meet selection criteria.
